# Contemporary Reproductive Outcomes for Patients With Polycystic Ovary Syndrome: A Retrospective Observational Study

**DOI:** 10.1210/jc.2015-2682

**Published:** 2016-02-09

**Authors:** D. Aled Rees, Sara Jenkins-Jones, Christopher L. Morgan

**Affiliations:** Pharmatelligence (S.J.-J.), Cardiff Medicentre, Heath Park, Cardiff CF14 4UJ, United Kingdom; and Institute of Primary Care and Public Health (C.L.M.) and Neurosciences and Mental Health Research Institute (D.A.R.), School of Medicine, Cardiff University, Cardiff CF24 4HQ, United Kingdom

## Abstract

**Context:**

Polycystic ovary syndrome (PCOS) is the most common cause of anovulatory infertility and may be associated with adverse pregnancy and neonatal outcomes. However, it is difficult to establish how much of this risk is due to PCOS and how much to obesity.

**Objective:**

This study aimed to determine the effect of PCOS upon fertility, pregnancy, and neonatal outcomes.

**Design and Setting:**

Data were extracted from the Clinical Practice Research Datalink (CPRD), a longitudinal anonymized primary care research database in the United Kingdom. Patients with a diagnosis of PCOS were matched to controls (1:2) by age (±1 y), body mass index (± 3 U), and CPRD practice. Standardized fertility ratios before and after diagnosis (index date) were calculated. Rates of miscarriage, pre-eclampsia, gestational diabetes, premature delivery, delivery method, and neonatal outcomes were compared.

**Results:**

Nine thousand sixty-eight women with PCOS matched study criteria. Prior to index date the standardized fertility ratio for patients with PCOS was 0.80 (95% confidence interval, 0.77–0.83); following index date it was 1.16 (1.12–1.20). The adjusted odds ratios (95% CI) for miscarriage (1.70; 1.56–1.84), pre-eclampsia (1.32; 1.16–1.49), gestational diabetes (1.41; 1.2–1.66), and premature delivery (1.25; 1.1–1.43) were all increased compared with controls. Of PCOS births, 27.7% were by Caesarean section compared with 23.7% of controls (1.13; 1.05–1.21). Infants born to mothers with PCOS had an increased risk of neonatal jaundice (1.20; 1.03–1.39) and respiratory complications (1.20; 1.06–1.37).

**Conclusions:**

PCOS is associated with subfertility but fertility rates are restored to those of the background population following diagnosis. Pregnancy complications and adverse neonatal outcomes are more prevalent for women with PCOS independently of obesity.

## Abstract (precis)

This study aimed to determine the effect of PCOS upon fertility, pregnancy, and neonatal outcomes. Fertility rates may be restored to those of the background population after diagnosis. Adverse pregnancy and neonatal outcomes are more prevalent for women with PCOS independently of obesity.

Polycystic ovary syndrome (PCOS) is the most common endocrine condition in women of reproductive age, affecting 5–10% of the premenopausal population (1, 2). The disorder is characterized by hyperandrogenism, insulin resistance, and glucose intolerance, which lead to an increased risk of type 2 diabetes (3). PCOS is also a common cause of anovulatory infertility (4, 5), such that many women require assisted reproductive techniques to help them conceive. Most commonly, this involves the use of clomiphene citrate as an agent to induce ovulation. However, two randomized, double blind, placebo-controlled trials have also established that metformin may improve ovulation and conception rates in clomiphene-resistant women with PCOS (6, 7). Previous studies have shown that the reproductive effects of the syndrome may also extend to a higher risk of many adverse outcomes in pregnancy, of which gestational diabetes (GDM), gestational hypertension, premature birth, and early pregnancy loss are the most studied (4, 5, 8, 9). Infants born to mothers with PCOS may also be at risk of adverse perinatal outcomes, including macrosomia, low Apgar score, and meconium aspiration (9). However, estimates of risk for these complications are difficult to ascertain due to heterogeneity in study design, small sample sizes, and inadequate matching for potential confounders such as obesity. In light of these uncertainties, we sought to describe the fertility outcomes and establish the relative risk of adverse pregnancy and neonatal outcomes in a large contemporary cohort of patients with PCOS in the United Kingdom.

## Materials and Methods

The study used a retrospective cohort design using data from the Clinical Practice Research Database (CPRD), a longitudinal, anonymized research database derived from more than 700 primary-care practices in the United Kingdom (10). Diagnostic information in CPRD is recorded using the Read code classification, a United Kingdom general practice standard. In addition, approximately 60% of those practices are linked to other English data sources including the Hospital Episode Statistics (HES) dataset. This provides data on all inpatient and outpatient contacts occurring within National Health Trusts.

### Patient selection and matching of controls

Women age 15–44 years with a diagnosis of PCOS defined by the Read code classification (Supplemental Table 1) or 10th revision of the International Statistical Classification of Diseases and Related Health Problems (ICD-10) classification (E28.2) between 2000 and 2012 were selected and defined as cases. The earliest diagnosis date was selected as the index date. A minimum “wash-in” period of 12 months from the patient's practice registration date to index date was used to maximize the likelihood that the case represented an incident case. Patients were followed until either leaving their practice or until the last CPRD collection data for their practice. Female controls were matched at a ratio of 2:1 by age (±1 y), body mass index (BMI) (± 3 U), and same primary care practice. All controls had to have remained at the same practice for at least the same duration from index date as their respective case, and followup was limited in both groups to that of the case.

### Outcomes and analysis

Baseline characteristics for cases and controls were presented and compared using univariate statistics (*t* test for continuous variables and χ^2^ for categorical variables).

Infertility was defined by relevant Read code (Supplemental Table 2) recorded for consultations within the primary care dataset. To assess the relationship between PCOS and infertility, time between first diagnosis of PCOS and first consultation for infertility was described graphically and by summary statistics. Fertility was compared between the two cohorts using standardized fertility ratios based on 10-year age bands using the non-PCOS population as the reference.

Miscarriages resulting in hospital admission were identified through secondary care sources. Crude relative risk of miscarriage was calculated. Multivariate logistic regression predicting miscarriage vs delivery was also performed adjusting for age, BMI, number of previous births, and smoking history.

Complications of pregnancy were defined as premature birth, GDM, and pre-eclampsia, and were ascertained from both the primary and HES data sources (Supplemental Table 3). Complications were assigned to each delivery if they were recorded in the preceding 295 days. The relative risks of each complication in the period, both before and after diagnosis of PCOS, were calculated. In addition, the odds ratio (OR) was calculated using multivariate logistic regression adjusting for age, BMI, multiple gestation, number of previous births, and smoking history.

To consider the effect of metformin treatment on pregnancy outcomes, date of conception was estimated as 280 days prior to delivery date for those pregnancies resulting in live birth. For the miscarriage outcome, it was assumed that conception occurred 84 days prior to the event. Metformin use was assumed based on prescription at two defined time points: 1) 90 days prior to estimated conception date and, 2) within the estimated first trimester of the pregnancy.

Hospital births were identified from the HES dataset. Delivery method was defined by procedural codes (OPCS-4) and classified as normal vaginal delivery, elective caesarean, emergency caesarean, forceps delivery, and vacuum delivery (Supplemental Table 4). Method of delivery and birth outcome was described and compared between cohorts using the χ^2^ test. In addition, multivariate logistic regression was used to examine the association of PCOS status with caesarean compared with vaginal delivery and the likelihood of twin delivery. Additional covariates included in the model were age, BMI, GDM, history of pre-eclampsia, and number of previous births. Length of stay, aggregated by delivery method, was compared using the *t* test.

Using the family number within CPRD, it was possible to link females with their children for a proportion of subjects. This allowed for diagnoses relating to neonatal admissions (defined as those occurring between birth and 7 d) to be compared. We selected the six most common groups of related complications on the neonate's inpatient record: jaundice (ICD-10 P580–P599), respiratory (P200-P229, P285, P288, and P289), feeding issues (P920–P929), overweight (P080, P081), low birth weight (P050–P059, P070–P072), and hypoglycemia (P703, P704). Analyses were adjusted for age, BMI, multiple gestation, number of previous births, and smoking history.

Studies using the CPRD are covered by ethics approval granted by Trent Multicenter Research Ethics Committee (Reference 05/MRE04/87). CPRD Independent Scientific Advisory Committee approval was granted for this study (ISAC 13–192).

## Results

**Figure 1.**
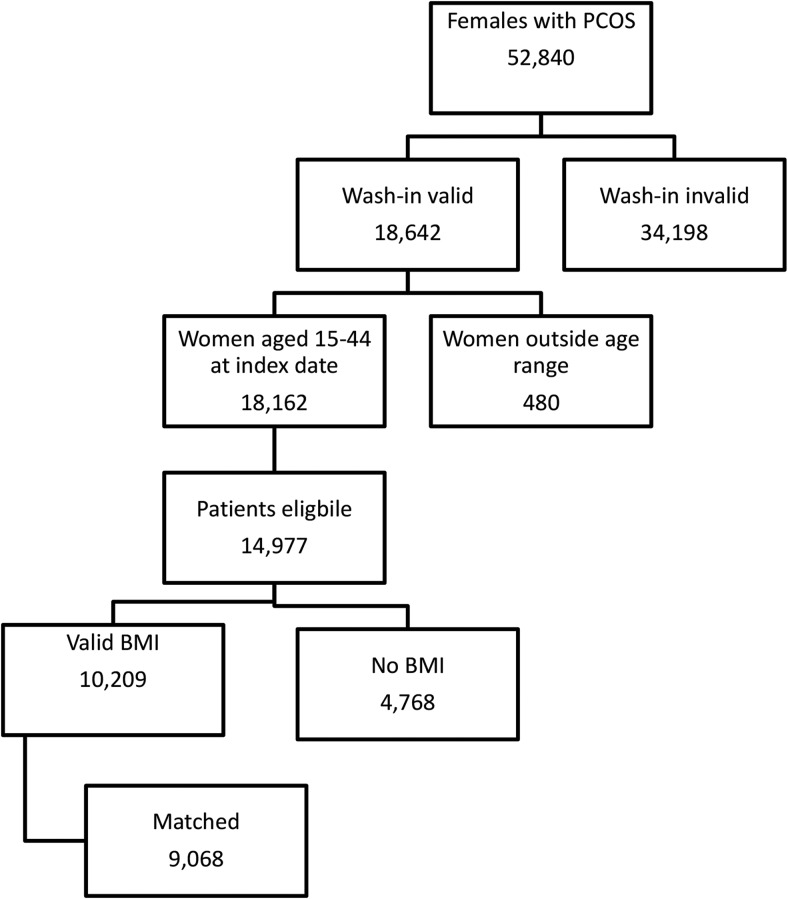
Patient selection.

Figure 1 shows the identification of PCOS patients, of whom 9068 could be matched to two non-PCOS controls. The baseline characteristics of these patients are shown in Table 1. There were significant differences between the PCOS and control cohorts for the number of primary care contacts (9.0 vs 7.6, respectively) in the previous year. There was considerable missing data for ethnicity, although this was lower for patients with PCOS compared with controls (33.0 vs 36.9%). There was a larger proportion of patients of Indian, Pakistani, or Other Asian origin compared with controls. Patients with PCOS were less likely to have ever smoked tobacco products (56.9 vs 52.9%).

**Table 1.** Baseline Characteristics for Patients With PCOS and Controls

Baseline Characteristic	Study Arm				*P*
	PCOS		Non-PCOS		
No. of patients	9068		18 136		
Followup (y), mean, sd	4.2	3.3	4.2	3.3	1.000
Observation period pre-index (y), mean, sd	5.3	3.7	6.5	3.8	<.001
Age (y), mean, sd	27.3	6.4	27.3	6.4	.808
Ethnicity					
Bangladeshi, n, %	27	0.3	60	0.3	<.001
Black (African/Caribbean/Other), n, %	174	1.9	331	1.8	
Chinese, n, %	13	0.1	22	0.1	
Indian, n, %	173	1.9	173	1.0	
Mixed, n, %	52	0.6	100	0.6	
Other Asian, n, %	77	0.8	83	0.5	
Other, n, %	103	1.1	202	1.1	
Pakistani, n, %	143	1.6	160	0.9	
Unknown/not recorded, n, %	2990	33.0	6701	36.9	
White, n, %	5316	58.6	10 304	56.8	
Primary care contacts in previous year	9.0	7.8	7.6	7.0	<.001
Prescribed oral contraception ± 90 d of index date, n, %	2712	29.9	6508	35.9	<.001
History of assisted reproduction, n, %	413	4.6	188	1.0	<.001
BMI (kg/m^2^), mean, sd	27.7	6.4	27.5	6.3	.019
Underweight (< 18 kg/m^2^), n, %	187	2.1	283	1.6	<.001
Normal (18–25 kg/m^2^), n, %	3543	39.1	7581	41.8	
Overweight (> 25–30 kg/m^2^), n, %	2330	25.7	4543	25.0	
Obese (> 30–40 kg/m^2^), n, %	2637	29.1	5013	27.6	
Extremely obese (> 40 kg/m^2^), n, %	371	4.1	716	3.9	
BP					
No. with BP recorded, n, %	5378	59.3	15 529	85.6	
Systolic BP (mm Hg), mean, sd	118.7	13.5	118.3	13.0	.046
Diastolic BP (mm Hg), mean, sd	74.8	9.7	73.5	9.6	<.001
Hypertensive, n, %	362	6.7	879	5.7	.001
Smoking status					<.001
Never smoked, n, %	5159	56.9	9590	52.9	
Ex-smoker, n, %	1511	16.7	3439	19.0	
Current smoker, n, %	2364	26.1	5065	27.9	
Not recorded, n, %	34	0.4	42	0.2	

Abbreviation: BP, blood pressure.

### Infertility consultations

**Figure 2.**
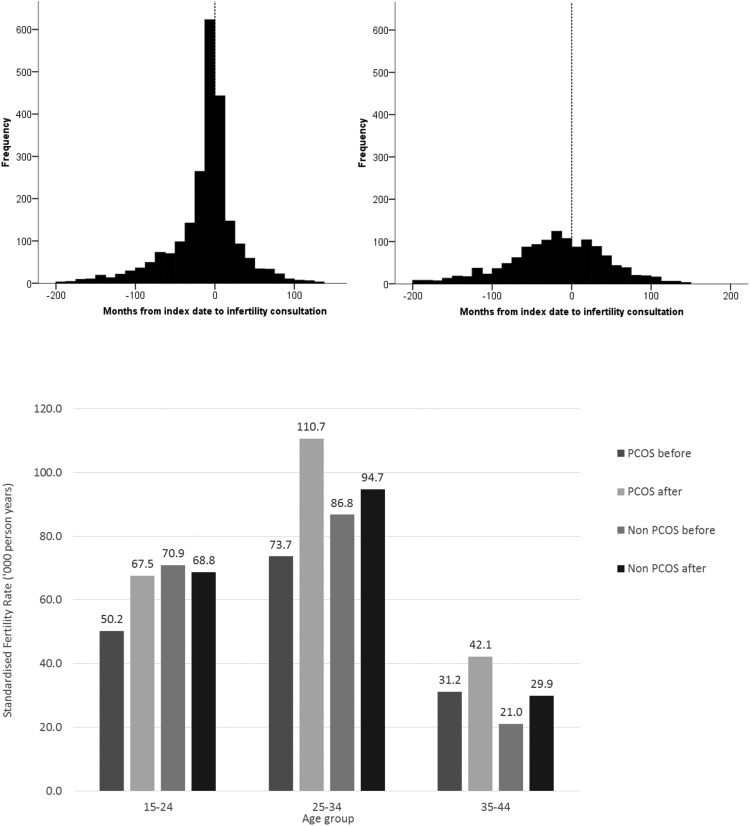
Upper panel: Time from index date (first diagnosis of PCOS) to consultation for infertility. Left upper panel: PCOS cases. Right upper panel: Non-PCOS cases. Lower panel: Standardized specific fertility rates for women with and without PCOS, before and after index date.

Of women identified with PCOS, 1529 (16.9%) had previously consulted their primary care practice regarding issues of infertility compared with 800 (4.4%) of controls; a crude rate ratio of 4.69 (95% confidence interval [CI], 4.30–5.11). Respective figures following PCOS index date were 796 (8.8%) vs 496 (2.7%); a crude rate ratio of 3.59 (3.21–4.02). Figure 2 (upper panel) shows time from index date to first consultation for infertility. Four-hundred twenty-three (18.2%) women with PCOS consulting their primary care practice for issues relating to infertility did so within ± 90 days of first PCOS diagnosis.

Of patients with PCOS, 413 (4.6%) had a history of assisted reproduction compared with 188 (1.0%) of controls. Following index date, 507 (5.6%) of cases had a record associated with assisted reproduction compared with 110 (0.6%) of controls. Subsequent to assisted reproduction, 286 (56.4%) of cases and 41 (37.3%) of controls had conceptions resulting in either hospital birth or miscarriage. Details of the fertility treatments provided are given in Supplemental Table 5.

### Age-specific fertility rates

During the study period (before and after index date) the overall fertility rate for women with PCOS was 67.3 per 1000 years compared with 70.6 for those without. The respective fertility rates pre- and postindex dates were 57.5 vs 71.8 and 79.8 vs 68.7. The age standardized ratios were 0.80 (95% CI, 0.77–0.83) prior to index date and 1.16 (95% CI, 1.12–1.20) after. Figure 2 (lower panel) shows the age-specific fertility rates by PCOS status before and after the index date.

### Pregnancy risks

**Figure 3.**
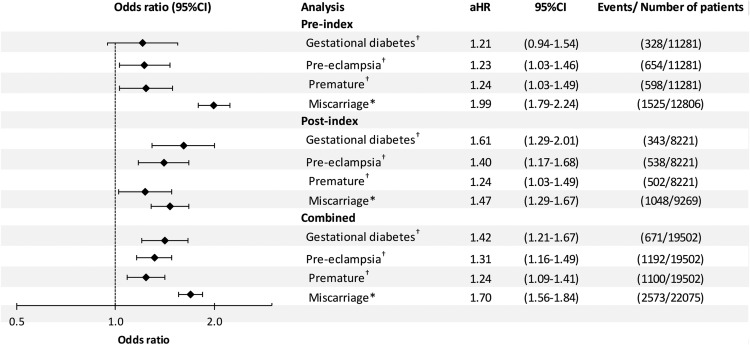
Adjusted^†^ ORs for GDM, pre-eclampsia, premature birth, and miscarriage for women with and without PCOS. *Denominator is all pregnancies, adjusted for age, body mass index, number of previous births, and smoking history.

There were 6861 pregnancies resulting in hospital admission for miscarriage or delivery for women with PCOS and 15 214 for those without (Table 2). Risk of miscarriage was increased for women with PCOS; the overall crude risk was 1.56 (95% CI, 1.45–1.68): 1.77 (1.61–1.95) before diagnosis and 1.35 (1.21–1.51) after. After adjusting for age, BMI, smoking status, and prior births, the OR was 1.70 (1.56–1.84). The respective ORs before and after index date were 1.99 (1.79–2.24) and 1.47 (1.29–1.67) (Figure 3).

**Table 2.** Hospital Admissions for Births, Miscarriages, and Complications of Pregnancy for Women With and Without PCOS

	PCOS		Non-PCOS	
	n	%	n	%
Before index date				
Identified pregnancies	3369		9437	
Births	2778	82.5	8503	90.1
GDM	93	3.3	235	2.8
Pre-eclampsia	188	6.8	466	5.5
Premature	171	6.2	427	5.0
Miscarriage	591	17.5	934	9.9
After index date				
Identified pregnancies	3492		5777	
Births	3021	86.5	5200	90.0
GDM	160	5.3	183	3.5
Pre-eclampsia	236	7.8	302	5.8
Premature	214	7.1	288	5.5
Miscarriage	471	13.5	577	10.0
Combined				
Identified pregnancies	6861		15 214	
Births	5799	84.5	13 703	90.1
GDM	253	4.4	418	3.1
Pre-eclampsia	424	7.3	768	5.6
Premature	385	6.6	715	5.2
Miscarriage	1062	15.5	1511	9.9

During those pregnancies resulting in live birth, the overall crude risk of pre-eclampsia was 1.31 (1.16–1.46). In the adjusted analyses, the OR was 1.31 (1.16–1.49); 1.23 (1.03–1.46) prior to diagnosis and 1.40 (1.17–1.68) afterward. The overall crude risk of GDM was 1.43 (1.23–1.67). In the adjusted analyses the OR was 1.42 (1.21–1.67); 1.21 (0.94–1.54) prior to diagnosis and 1.61 (1.29–2.01) afterward. Premature delivery was also increased for women with PCOS. The overall crude risk was 1.27 (1.13–1.44). After adjusting for other factors, the OR was 1.24 (1.09–1.41); 1.24 (1.03–1.49) prior to diagnosis and 1.26 (1.05–1.52) afterward (Figure 3). In a sensitivity analysis restricted to singleton births there was little difference in the adjusted ORs compared with the main analysis (Supplemental Table 6). In a further sensitivity analysis restricted to the 4355 patients with a diagnosis of either PCOS or Stein-Leventhal syndrome and their respective controls, slightly higher adjusted ORs were observed for all outcomes (Supplemental Figure 1).

### Effect of metformin

For the analysis restricted to PCOS patients treated with metformin relative to other nonmetformin treated patients, there was a significant increase in pre-eclampsia associated with metformin use in both the 90 days prior to estimated conception (OR, 1.54; 95% CI, 1.06–2.23) and the first trimester of pregnancy (OR, 1.54; 95% CI, 1.07–2.22) but no significant difference in any other pregnancy related outcomes (Supplemental Figure 2).

### Delivery methods

Supplemental Table 7 shows the method of delivery for women with and without PCOS. Of PCOS births, 1606 (27.7%) were by caesarean section compared with 3243 (23.7%) of non-PCOS. In logistic regression, the OR of caesarean delivery for PCOS was 1.13 (1.05–1.21) after adjustment for other covariates including pre-eclampsia (2.54; 2.14–3.00) and GDM (2.63; 2.25–3.06). Mean length of stay for delivery was significantly greater for women with PCOS (3.8 vs 3.5 d; *P* = .002; Supplemental Table 7). After adjustment for age, BMI, GDM, pre-eclampsia, and premature delivery, the respective means were 3.7 vs 3.5 days (*P* < .001).

### Neonatal outcomes

Delivery outcome was available for 5757 (99.3%) PCOS births and 13 607 (99.3%) non-PCOS births (Supplemental Table 8). Multiple births occurred in 146 (2.5%) of PCOS births and 226 (1.7%) of non-PCOS births, an increased relative rate for mothers with PCOS (OR, 1.54; 95% CI, 1.22–1.95).

Apgar scores (11) were available for 1731 (30.1%) children born to mothers with PCOS and 4217 (31.0%) born to mothers without PCOS. Respective Apgar scores were 6.33 and 6.43 at 1 minute and 6.97 and 7.02 at 5 minutes, and were not significantly different between groups.

Neonatal inpatient admissions records for children could be linked to 3634 (62.7%) of births for women with PCOS and 8557 (62.4%) of those without. Table 3 shows the number of defined neonatal complications recorded on these admissions. There were significant increases for jaundice (1.20; 1.03–1.39) and respiratory complications (1.20; 1.06–1.37).

**Table 3.** Neonatal Complications Reported for Offspring of Women With PCOS and Matched Controls

Complication	PCOS (n = 3707)		Non-PCOS (n = 8656)		Adjusted OR^a^ (95% CI)	*P*
	n	%	n	%		
Jaundice	326	8.8	588	6.5	1.20 (1.03–1.39)	.016
Respiratory	420	11.3	785	8.7	1.20 (1.06–1.37)	.005
High birth weight	40	1.1	104	1.2	0.97 (0.67–1.41)	.894
Low birth weight	242	6.5	439	4.9	1.19 (1.00–1.42)	.054
Hypoglycemia	124	3.3	218	2.4	1.31 (0.99–1.74)	.062
Feeding issues	85	2.3	139	1.5	1.21 (0.96–1.52)	.106

Abbreviation: OR, odds ratio.

a Adjusted for age, BMI, number of previous births, multiple gestation, and smoking history.

## Discussion

In this large population-based study, a diagnosis of PCOS was associated with a lower fertility rate, increased risk of adverse pregnancy and neonatal outcomes, and of operative delivery, which were not attributable to obesity.

We observed an approximate 4-fold increase in consultations for infertility in women with PCOS compared with matched controls. This is reflected in lower fertility rates for women with PCOS prior to diagnosis, particularly in younger patients. This may reflect a more severe phenotype presenting at this age, but is also consistent with a trend toward regularization of the menstrual cycle in women with PCOS with advancing age (12). After diagnosis, fertility rates were restored in all age groups to those of the background population, suggesting that infertility in women with PCOS is eminently treatable. However, it is not possible from the data to compare the intention to conceive between females with and without PCOS.

We also confirmed an increased risk of miscarriage in women with PCOS. Only a few studies have examined the association between PCOS and early pregnancy loss (13–17). Some of these suggested that the risk of spontaneous abortion was higher in women with PCOS but only one study adjusted for obesity and fertility treatment (13) which are themselves known to be associated with an increased risk (18). In our study, which matched patients for BMI, PCOS was associated with a significant increase in pregnancy loss both before and after PCOS diagnosis.

For those pregnancies resulting in live birth there was also significant increases in pre-eclampsia, GDM, and premature delivery. Previous studies have suggested that the risk of GDM is increased in women with PCOS, but interpretation of these studies and estimates of risk are difficult because of differences in study design and small sample sizes. One population-based study estimated a 2.4-fold increased risk of GDM in women with PCOS compared with women without a diagnosis of PCOS or symptoms (19). However, the PCOS group was significantly older and had a higher prevalence of multiple gestation than controls, which themselves are risk factors for GDM. Furthermore, given that groups were not matched for obesity, it is unclear how much of these risks were due to PCOS per se and how much to obesity, which is common in this patient population and is itself associated with a higher risk of GDM. Indeed, one previous study showed that the prevalence of GDM did not differ among women with PCOS and controls when weight was matched for (20). In contrast, Roos et al (9), in a large population-based study of singleton births in Sweden, found that the risk of GDM was more than doubled in women with PCOS, even after adjustment for age, BMI, and use of assisted reproductive technology. This resonates with our understanding of PCOS as a metabolic disorder underpinned by defects in insulin secretion and sensitivity. Our observations are consistent with these findings, albeit that the OR was slightly lower at 1.4. This is also in keeping with our previous CPRD study, which showed that women with PCOS had a 50% increased risk of type 2 diabetes in the United Kingdom population (3).

Our finding of an increased risk of pre-eclampsia is consistent with many (4, 21), but not all (22) previous studies but the mechanisms by which this develops are unclear. One such pathway may involve hyperinsulinemia, which is a common finding even in lean patients with PCOS, and which has been implicated in hypertension developing in pregnancy (22, 23).

Two meta-analyses assessing pregnancy outcomes in women with PCOS have suggested that the risk of preterm delivery is increased in patients with the syndrome (4, 24). Of the studies that informed these analyses (5, 20, 22, 25, 26), only one excluded patients with multiple gestation (26), which is a major risk factor for preterm delivery in its own right. Mikola et al (22) found that PCOS per se lost its significance as a risk for preterm birth in a multivariate analysis which identified multiple gestation and nulliparity as independent predictors. This may be in keeping with the observation that many women with PCOS need ovulation induction to conceive and that such treatment is more likely to result in multiple gestation pregnancies. We found an increased risk of premature delivery in our population, which could not be explained by differences in age, BMI, multiple gestation, or previous pregnancy. This observation is in keeping with that of Roos et al (9) in their study of singleton pregnancies. However, obesity may exacerbate the risk of preterm birth. De Frène and colleagues (27) reported an almost seven-fold increase in the proportion of preterm deliveries when comparing obese and nonobese females with PCOS.

The effect of metformin use for women with PCOS was inconclusive as this study was not powered to detect differences within the PCOS cohort and the effect of multiple testing should be considered in interpreting these results. However, there was a significant increase in pre-eclampsia for those women identified as being prescribed metformin prior to estimated conception and during their first trimester. It is difficult to be certain whether this represents a true adverse effect of metformin therapy or is more likely a reflection of residual confounding due to preferential prescribing in higher risk pregnancies. A large, multicenter randomized clinical trial found no effect of metformin, administered late in the first trimester through to delivery, on pregnancy outcome, with the exception of an apparent reduction in later miscarriage and preterm delivery (28). We did not find an effect of metformin on early miscarriage rate, consistent with previous studies (29, 30).

In our study we have also shown that caesarean section was more common as a method of delivery in patients with PCOS, even after adjustment for possible confounders. This is in agreement with some (9) but not all studies (4). Indeed, in a meta-analysis of pregnancy outcomes in women with PCOS where subgroup analysis was restricted to higher validity studies, no increased risk was observed (4). An increased rate of operative delivery was associated with a 6% increased length of stay in fully adjusted analyses. This is likely to lead to adverse health economic consequences, of a similar order of magnitude as that apparent in obesity (31). As anticipated, a diagnosis of PCOS was also associated with an increased risk of multiple births likely related to a greater use of assisted conception, albeit that multiple births only accounted for a small fraction of the total.

Neonatal outcomes were generally worse for infants born to mothers with PCOS although we did not find a difference in stillbirth nor in Apgar scores with controls. This contrasts with Roos et al (9), who found an increased risk of low Apgar score at 5 minutes in infants born to mothers with PCOS but no increased risk of neonatal death. However, we did note a significantly increased risk for neonatal jaundice and respiratory distress. To our knowledge, ours is the first study to report such associations. There was also a trend toward increased risk of low birth weight in our study, a finding which has been shown in one (32) but not other previous studies (9, 20, 22, 33), and also hypoglycemia.

Significant strengths of our study include the large sample size and careful adjustment for possible confounders, but our study has certain limitations. In common with other studies of this type, the CPRD collects data from routine practice; thus, there are missing data and coding imperfections. However, whereas these may introduce noise into the study, they are unlikely to introduce bias. We were dependent upon hospital data for the accurate ascertainment of births and therefore births outside National Health Service hospitals (private or home births) could not be included. Home births represent approximately 2.5% of all births in the United Kingdom and it is possible that there are differences in this proportion for those with and without PCOS due the perceived risk of complications. Similarly, we did not have access to data for infertility consultations that occurred in the private sector. We were unable to analyze the effect of PCOS phenotype on reproductive outcome given that these data are not captured in routine primary care. Based on routine data, we were also unable to determine the proportion of women in each group who were seeking to become pregnant. Within the PCOS group, fewer women were prescribed contraceptives within ± 90 days of index date. It is also noticeable (Figure 2Ai) that for women with PCOS, first consultations for infertility are not uniformly distributed but centered around first diagnosis of PCOS. This may be due to PCOS being diagnosed during investigations for infertility, which may be indicative that the patient is seeking to become pregnant.

In conclusion, we have shown that younger women with PCOS have reduced fertility but that fertility rates may be restored to those of the background population after diagnosis. We have also confirmed an adverse effect of PCOS, independently of obesity, on several pregnancy outcomes, including GDM, pre-eclampsia, and preterm birth. These observations suggest that women with PCOS require more intensive monitoring during pregnancy, and emphasize the need for additional studies to understand the mechanisms by which these complications arise.
